# The Unqualified Assistant

**Published:** 1889-09-14

**Authors:** 


### The Unqualified Assistant.

Of the student class, yet not a student, sharing the responsibilities of the practitioner, while yet his education is incomplete, and picking up practical knowledge apart from its scientific basis, the unqualified assistant stands out as a unique, lonely, and rather pathetic figure. Pathetic—whether his temporary absence from the classroom and the hospital be occasioned by good reasons or the reverse. He belongs to one of two classes: either he is one of those who, conscious of vocation, set themselves to win a medical degree against all obstacles of fate and fortune, or he is one of those who, having thrown away the opportunities set before them, are forced into retirement by needless poverty, there to meditate on time and money misspent. The first type is decidedly the more estimable. Whatever we may think of the employment of unqualified assistants, we must admire the man who makes so brave a struggle to attain the object of his ambition, who goes on undaunted, living, both physically and intellectually, from hand to mouth, utilising his knowledge as he acquires it, though sometimes attempting work beyond his powers. How hardly his degree is won none but himself can know. It may be surmised that hunger is not altogether unfamiliar to him, and minor privations have become second nature. It is quite possible that his principal may not be the most considerate of men, and it is more than likely that his principal's wife will look askance on his shabby coat, and the *gaucherie* of manner that springs from preoccupation. His life at the best is not an easy one. The assistant naturally gets the hardest work to do—the long journeys, the weary waiting in poverty-stricken cottages, the encounters with that most exacting of beings, the club patient. Worst of all is the scantiness of leisure, which makes it all but impossible to keep up the scientific knowledge he has already acquired, and keeps him in a chronic state of dread lest by the time he has earned the guineas necessary for his examination the knowledge demanded in it shall have left him.

The other type is more pleasing at first. Cheerful, good-natured, and ready-witted, the patients like him; the principal thinks he may venture to take a holiday now that he has such a "gentlemanly fellow" to take his place, and it is said in the district that Dr. So-and-so's new assistant is quite an acquisition to society. But Dr. So-and-so is not so well pleased to find, when he comes back from that brief vacation, that his assistant has spent half his afternoons at tennis parties, and that one of his best patients has gone to the rival practitioner. Perhaps the vexation is of a more serious nature. The assistant appears one day with dull eyes and an unsteady hand, and it dawns upon those who have hitherto liked him best that the interruption to his medical studies may come from rather discreditable causes. Perhaps the *désillusionnement* comes in some still more serious form. A case treated in rash, hap-hazard fashion, an operation foolishly undertaken and badly done, a life imperilled or lost; and the pleasant assistant disappears suddenly from the neighbourhood, while the principal goes about with an anxious countenance, making, it is to be hoped, vows against the employment of unqualified men in future.

For, though exceptions may be found now and then, it can never be safe for any man to undertake the varied responsibilities of practice until he has gone through the whole course of medical study. It may be a myth, that story of the student who told a patient to come back in a week or two, because he seemed to be suffering from a fever, and "our class has only got as far as fits." But it only exaggerates the truth that at any period of his education a man may find himself confronted with a problem he has not yet been taught to solve. Once he has gone through his curriculum, an assistantship in a country practice will do him no harm. It will teach him to apply on his own initiative the knowledge he has acquired, and will bring all his intelligence to the front when he has to work without the manifold appliances and ideal surroundings of hospital practice. The hardness of the experience may dispel certain rose-coloured dreams he has cherished about a doctor's life; but this is not wholly to be deplored. Only it is necessary, for the sake of his patients, that he should have learned all his college can teach him, that he should not be in any sense, either technically or practically, an "unqualified assistant."

